# Cancer exploits a late premetazoan gene module conserved in the human genome

**DOI:** 10.1016/j.gendis.2023.01.020

**Published:** 2023-03-28

**Authors:** Vladimir F. Niculescu

**Affiliations:** Kirschenweg 1, Diedorf 86420, Germany

From an evolutionary perspective, the human cancer genome is based on an ancient gene module developed by the common ancestor of the Amoebozoa, Metazoa, and Fungi (AMF).^1^ The transition phase to multicellularity (premetazoan period) was a rather long period of evolutionary attempts and failures. During this time, suppressor genes were evolved to deactivate the G + S cell system inherited from the AMF ancestor, and anti-suppressor genes evolved to reactivate it in case of failed evolutionary attempts. These suppressor and anti-suppressor genes were the archetypes of tumor suppressors and oncogenes. Genes from the failed evolutionary attempts were not discarded but added as remnants to the so-evolved *premetazoan genome*. During the subsequent evolution of metazoans and animals, the reactivation of the premetazoan genome generates cancer. Animal cancers evolved many specific anti-host genes. The *human cancer genome* consists of (i) founder germ and soma G + S genes belonging to the AMF cell system, (ii) evolutionary failed gene packages from the premetazoan evolution,^2^ and (iii) specific anti-host genes evolved during animal evolution (specific anti-host genes) (Fig. 1). All these gene packages and regulatory gene networks generate the phenotypic heterogeneity of cancer and tumors.

**Figure 1:**
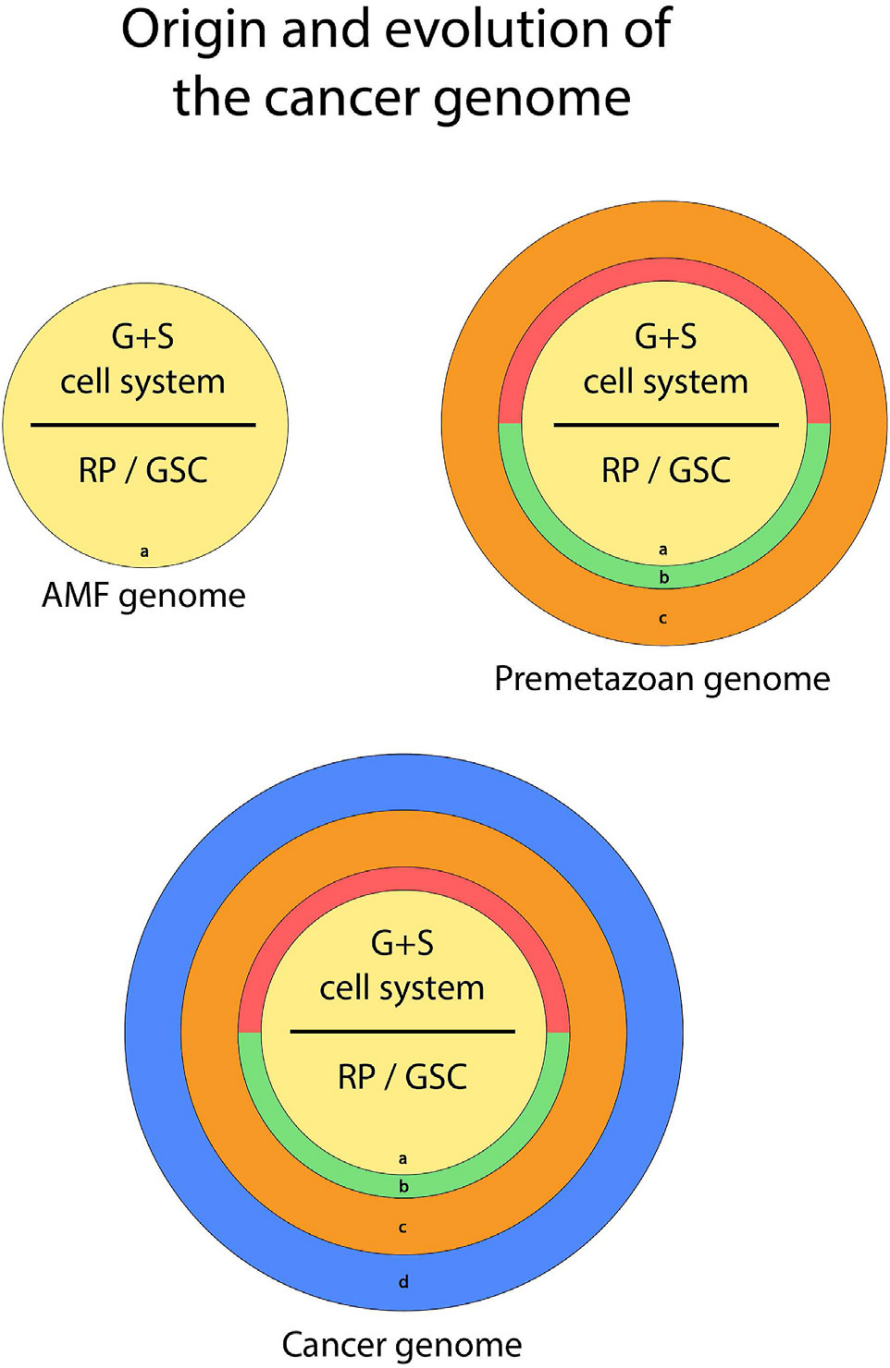
Cancer uses a reminiscent premetazoan genome embedded in the genome of all metazoans including humans. All metazoans inherited the basic G + S genome from the common AMF ancestor (a, yellow) including the basic cell biological features of carcinogenesis (CSCs-forming germlines, polyploidization, ACD phenotype, oxygen sensitivity, germline damage, and genome reconstruction, PGCCs, EMT, and MET).^1^^,5^ The first attempts at multicellularity required a suppressor gene network against the AMF genome (b, red), but also anti-suppressor genes (b, green) to return to the G + S system in case of dead ends. Genes from those failed evolutionary attempts, which were not involved in further approaches, were not discarded but stored in the genome of the premetazoans (c, brown). During the evolution of metazoans, which was repeatedly accompanied by cancer outbreaks, the pre-metazoan gene module co-evolved and was enriched by numerous anti-host genes (d, blue).^1^^,^^2^

During the evolution of metazoans, host organisms developed so-called hub genes,^2^ which act as a kind of seesaw between the various AMF and pre-metazoan genes on the one hand and the multicellular (metazoan) genes on the other. They ensure a functioning balance between premetazoan and multicellular genes and a stable equilibrium in healthy organisms. In cancer, the balance tips in favor of premetazoan genes.

The human genome contains even more genome remnants from the deep past that are in a state of facultative or partial reactivation. Human endogenous retroviruses (HERV) which make up around 8% of the human genome, were left behind as a result of infections that primate ancestors suffered many My ago. Particularly interesting, HERV genes are active in tumors, and infections, as well as during embryonic development.^3^

## Amoebozoa and Metazoa – the sister clades

Great help in clarifying the evolutionary history of the cancer genome and the cancer life cycle came from the life cycle biology of amoebozoans,^4^ which are closer to the common AMF ancestor and could clarify many cancer life cycle features. As homologous “sister life cycles”, the G + S cell systems of cancer and *Entamoeba* help each other clarify their roots. The life cycle of amoebae helps to understand the life cycle of cancer, and conversely, cancer cell biology helps to better understand the amoeba life cycle. It could be clarified that the G + S life cycle originated in the AMF common ancestor and was inherited from the branching Amoebozoa and Metazoa clades. Last but not least, it could explain the immortality of cancer and its sophisticated cell system inherited from the AMF common ancestor.

## The germ and soma (G + S) cell system

The homology of the two G + S cell systems of cancer and amoebozoa (amoebae) is striking. Both consist of an oxygen-sensitive germline capable of producing stem cells via asymmetric cell cycles and reproductive RP/GSC cycles that generate germline stem cells (GSCs).^1^ Oxygen-damaged germline cells cannot produce stem cells and must be replaced either by (i) soma-to-germ transition (SGT, in cancer EMT) or by (ii) cell and nuclear fusion and genome reconstruction. This occurs in the polyploid giant nuclei of multinucleate genome repair syncytia MGRS, called PGCC in cancer.

In contrast to the ancient germline, somatic cell lines are evolutionarily younger. They evolved at a time when more oxygen was available in the environment. The somatic cell line is oxygen resistant and maintains the integrity of the genome. It can regenerate the germline through SGT/EMT processes and boost the production of GSCs/CSCs. That explains why CSCs can arise from non-CSCs.^4^ Cancer is a disease common to all metazoans. It is the never-ending battle of a therapeutically resistant relic genome against a misconceived dead-end cell community. The cancer genome evolved in metazoans through specific anti-host genes, in parallel with the evolution of metazoans and host organisms.

## The ubiquity of the AMF germline

The G + S cell system (or parts thereof) is widely distributed in metazoans. Germline genes are expressed not only in cancer but also in gametogenesis, ovogenesis, and embryogenesis in both invertebrates and vertebrates, and mammals. They provide embryonic and adult stem cells responsible for tissue differentiation and organogenesis. Cancer does not arise from adult stem cells, as previously thought. Similarities between cancer repair mechanisms such as PGCCs and embryonic structures in humans and mammalians suggest a deep homology of cancer and embryonic cell structures to the common AMF ancestor, but not a causal relationship (origin) between cancer and embryonic cell structures.

## From premetazoan life preserver to metazoan life destroyer

One of the fundamental questions related to cancer genetics is why the founder genes of the AMF ancestors and the failed genes from the premetazoan era were not discarded, but have been retained in the metazoan and human genomes. The reason for this lies in genome economics: life does not discard anything that gives it a current advantage or can be used for evolutionary progress. This strategy not only brought significant evolutionary advantages but also opened the door to disease and cancer.

Premetazoan cell systems that dared to go the way of multicellularity but missed it, relied on the AMF life cycle as a lifeline and survived the evolutionary dead end. It got the chance to make further evolutionary attempts. The constant back and forth ⁠— one step forward, half a step back — ensured that the G + S cell system itself remained reactive and the AMF genome experienced tremendous gene enrichment. The premetazoan period was a time of hidden nonlinear evolution.^2^

The additional genes that were retained from the failed evolutionary attempts expanded the genetic portfolio of premetazoans (Fig. 1). The additional genes enable the premetazoan genome — when reactivates in metazoans — to higher invasive potential, increased phenotypic diversity, increased capacity for cell fusion, and other traits typical to cancer. The G + S cell system of cancer has more potential. It cannot be ruled out that both G + S cell systems — of cancer and amoebozoans — have basic AMF genes that favor pathogenicity. In cancer, however, the virulence of the G + S cell system is directed against its host organism. With the evolutionary transition to multicellularity, the G + S cell system changes from a life sustainer to a life destroyer. Reactivation of the old premetazoan genome destroys both the host and the pathogen.

## Conflict of interests

The author declares no conflict of interests.
